# Droplet spatial distribution of oil-based emulsion spray

**DOI:** 10.3389/fpls.2023.1183387

**Published:** 2023-06-08

**Authors:** Chen Gong, Fujun Chen, Bingbo Cui, Aichen Wang, Zhao Zhang, Zhenjiang Zhou, Yufei Liu

**Affiliations:** ^1^ School of Agricultural Engineering, Jiangsu University, Zhenjiang, China; ^2^ College of Information and Electrical Engineering, China Agricultural University, Beijing, China; ^3^ College of Biosystems Engineering and Food Science, Zhejiang University, Hangzhou, Zhejiang, China

**Keywords:** oil-based emulsion spray, sheet structure, droplets distribution, nozzle configuration, emulsion concentration

## Abstract

**Introduction:**

Oil-based emulsion solution is a common pesticide formulation in agricultural spraying, and its spray characteristics are different from that of water spraying. The well understanding of its spray characteristics is the theoretical basis to improve the pesticide spraying technology. The objective of the present study is to deepen the understanding of the spray characteristics of oil-based emulsion.

**Method:**

In this paper, the spatial distribution characteristics of spray droplets of oil-based emulsion were captured visually using the high-speed photomicrography. On the basis of image processing method, the droplet size and distribution density of spray droplets at different spatial locations were analyzed quantitatively. The effects of nozzle configuration and emulsion concentration on spray structures and droplet spatial distribution were discussed.

**Results:**

Oil-based emulsion produced a special perforation atomization mechanism compared with water spray, which led to the increase of spray droplet size and distribution density. Nozzle configuration had a significant effect on oil-based emulsion spray, with the nozzle changed from ST110-01 to ST110-03 and ST110-05; the sheet lengths increased to 18 and 28 mm, respectively, whereas the volumetric median diameters increased to 51.19% and 76.00%, respectively. With emulsion concentration increased from 0.02% to 0.1% and 0.5%, the volumetric median diameters increased to 5.17% and 14.56%, respectively.

**Discussion:**

The spray droplet size of oil-based emulsion spray can be scaled by the equivalent diameter of discharge orifice of nozzles. The products of volumetric median diameters and corresponding surface tensions were nearly constant for the oil-based emulsion spray of different emulsion concentrations. It is expected that this research could provide theoretical support for improving the spraying technology of oil-based emulsion and increasing the utilization of pesticide.

## 1 Introduction

Oil-based emulsion, as one of common formulation for plant protection, is widely used in agricultural spraying (Li et al., 2022; Liu et al., 2022). The oil-based emulsion spray was supposed to have a same atomization mechanism with water spray over a period of time. However, in recent years, many research studies indicated that oil-based emulsion spray has a significantly different spray characteristics compared with water, and it causes a different droplet size distribution that plays a key role in agricultural application (Gong et al., 2020; Gong et al., 2021; Gaillard et al., 2022). Water spray has been widely studied in both experiments (Lewis et al., 2016; Kooij et al., 2018; Gong et al., 2020; Gong et al., 2022) and theories (Post and Hewitt, 2018; Qin et al., 2018); however, the work related to oil-based emulsion spray is limited.

For water spray, it is pointed out that its atomization process can be generally divided into two steps: the development of ripple structure on the spray sheet that leads to the formation of ligaments (Altieri et al., 2014; Qin et al., 2018; Li et al., 2021), followed by the fracture of ligaments and the formation of droplets due to interface instability (Qin et al., 2018). These include Rayleigh–Taylor instability (liquid sheet to ligament) and Rayleigh instability (ligament to droplet) mechanisms (Makhnenko et al., 2021). The spray droplets also undergo a series of spatial evolutionary processes before reaching the target (Lewis et al., 2016). Scholars had studied the droplet size distribution in terms of spray pressure, nozzle type, and spray space locations by methods such as numerical simulation (Musiu et al., 2019) and mathematical modeling (Dwomoh et al., 2020; Chen and Ashgriz, 2022; He et al., 2022). The result indicated that an increase in working pressure caused an increase in droplet pulverization regardless of the type of nozzle. In different spray pressure and orifice diameter values, the droplet volume percentage that presents a normal distribution, according to the mathematical model, can well predict the droplet size distribution. 

For oil-based emulsion spray, the oil-phase particles lead to the generation of perforations in the spray sheet and the different breakup mechanism (Qin et al., 2010; Makhnenko et al., 2021). The development of perforations causes the earlier breakup of spray sheet (Gong et al., 2020; Cryer et al., 2021; Li et al., 2021). Compared with water spray, the droplet of oil-based emulsion spray has a larger average size and a narrower size distribution (Qin et al., 2010; Vieira et al., 2018; Gong et al., 2020). The droplet size distributions of water spray are commonly studied by using laser diffraction (LD) and phase Doppler particle analyzer (PDPA) methods (Vernay et al., 2016; Vernay et al., 2017; Vieira et al., 2018; Sijs and Bonn, 2020; Li et al., 2021; Zhang and Xiong, 2021). The LD method is based on light diffraction, and it measures droplet size by Mie theory with complete or fuzzy approximation (ISO13320:2009) (De Cock et al., 2016). The PDPA method measures the size and velocity of droplets based on light scatter (Blaisot and Yon, 2015). These non-contact measurement techniques are based on optics diagnostics (Blaisot and Yon, 2015). However, for the emulsion spray, the transmittance of droplets is limited; therefore, the above methods are supposed to bring errors (Tuck et al., 1997; Hilz and Vermeer, 2013). The image processing method is more suitable for measuring those droplets that have relatively low transmittance (Zeng et al., 2015; Farzad et al., 2017; Patil et al., 2017). The droplet sizes in the spray image can be accurately measured on the basis of the edge gray intensity criterion (De Cock et al., 2016; Minov et al., 2016).

In this paper, the oil-based emulsion spray is focused, and its sheet structure and spray droplets of different spatial locations were captured using high-speed photomicrography technology. An image processing method was used to quantitatively measure the spatial distribution of the spray droplet size and distribution density. The relationship between spray sheet structure and spatial distribution of spray droplets was investigated. In addition, the effects of nozzle configuration and emulsion concentration on spray structures and droplet spatial distribution were discussed. It is pointed out that this research could help to deepen the understanding of the spray characteristics of oil-based emulsion and provide a theoretical support for improving the spraying technology of oil-based emulsion and increasing the utilization of pesticide.

## 2 Materials and methods

### 2.1 Experimental setup

**Figure 1:**
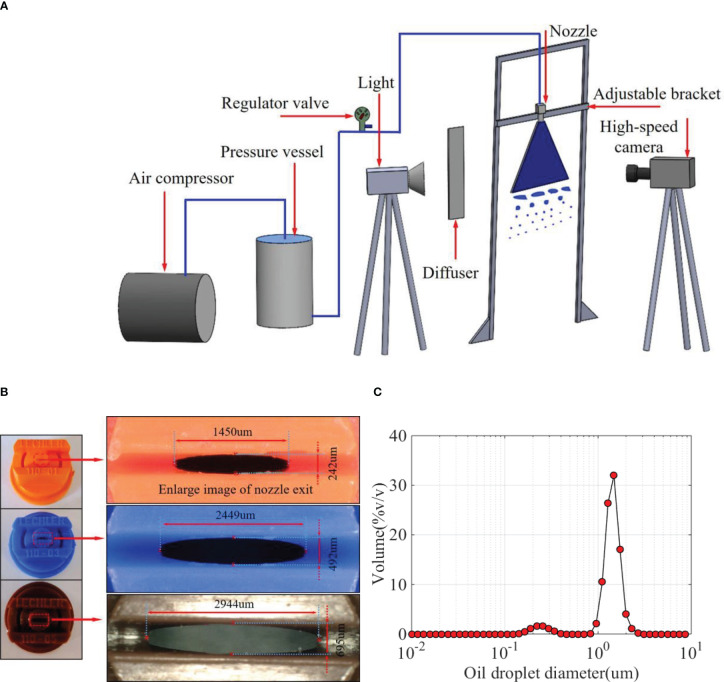
Spray experimental setup. **(A)** Experimental setup and **(B)** nozzles and discharge orifices. **(C)** Size distribution of oil droplets in oil-based emulsion solution.

The main experimental devices are shown in **Figure 1A**. The spray liquid was stored in the pressure vessel (the capacity of the vessel is 10 L) and was pressurized by an air compressor. A pressure regulator valve (SMC AR-3000, SMC China Co., Shanghai, China) was used to control the spray pressure, and its precision is 0.02 MPa. The spray pressure was set as 0.3 MPa. The nozzles are fixed on an adjustable bracket, which can move in the vertical direction. The precision of the movement is 1 mm. A high-speed camera (Olympus I Speed 3, Olympus Co., Shinjuku-ku, Tokyo, Japan) was used to capture the spray structures of different vertical positions. A light and a diffuser are used to produce a uniform backlight. As indicated in **Figure 1B**, three standard flat-fan spray nozzles—ST110-01, ST110-03, and ST110-05 (Lechler Inc., Metzingen Germany)—were used in the experiments. The long diameter ( $D_{L}$ ) and short diameter ( $D_{S}$ ) of the nozzle exit are measured using a microscope (VHX-900F, Keyence Co., Japan). As indicated in **Figure 1B**, the exit of the nozzles has elliptical structures, and it is not suitable to use long diameter or short diameter to characterize the size of nozzle exit. Therefore, the equivalent diameter (Duarte and Corradini, 2018) $D_{E}=\sqrt{D_{L}D_{S}}$ was used to characterize the size of nozzle exit. The detail results are presented in **Table 1**.

**Table 1:** The structural parameters of the nozzle exit.

Nozzle	Long diameter $D_{L}$ /μm	Short diameter $D_{S}$ /μm	Equivalent diameter $D_{E}$ /μm
ST-110-01	1450	242	592.37
ST-110-03	2449	492	1097.68
ST-110-05	2944	695	1430.41

Both water and oil-based emulsions were used as the spray liquid in the experiments. The oil-based emulsion was prepared with pre-emergent herbicide butachlor (Jiangsu Lvlilai Co., Kunshan, Jiangsu, China) and water. The main composition of the herbicide butachlor was as follows: 6% w/w sodium alkylbenzenesulfonate (emulsifiers), 9% w/w Styrylphenyl polyoxyethylene ether (emulsifiers), 15% w/w cyclohexanone (solvent), and 60% w/w (butachlor). The size distribution of oil droplets in oil-based emulsion solution in the experiment was measured using the Malvern particle size analyzer (type: Zetasizer Nano ZS90), as shown in **Figure 1C**. Some research studies indicated that oil-based emulsion has a significant effect on the spray droplet size as the emulsion concentration is in the range of 0.05%–0.6% (Dexter, 2001; Vernay, 2015). Therefore, the emulsion volume concentrations of 0.02%, 0.1%, and 0.5% were selected in the experiments to evaluate the effect of emulsion concentration on the spatial distribution of spray droplets. The static surface tension of different spray medium was measured using a contact angle measurement system (KSV CAM101, KSV Instruments, Ltd., Helsinki, Finland). The experimental conditions were indoor, and the room temperature was 23°C–25°C. Six groups of experiments were designed. Groups 1 and 2 were used to discuss the difference between water and oil-based emulsion sprays. Groups 2 to 4 were used to evaluate the effect of emulsion concentrations on the oil-based emulsion spray. Group 2, 5, and 6 were used to evaluate the effect of nozzle configuration on the oil-based emulsion spray. The parameters of different experimental conditions are listed in **Table 2**.

**Table 2:** Experiment conditions.

Test number	Nozzle	Pressure P (MPa)	Concentration	Surface tension σ (N m^−1^)
1	ST110-03	0.3	0	0.070
2	ST110-03	0.3	0.1%	0.041
3	ST110-03	0.3	0.02%	0.050
4	ST110-03	0.3	0.5%	0.032
5	ST110-01	0.3	0.1%	0.041
6	ST110-05	0.3	0.1%	0.041

### 2.2 Capture of spray structures

**Figure 2:**
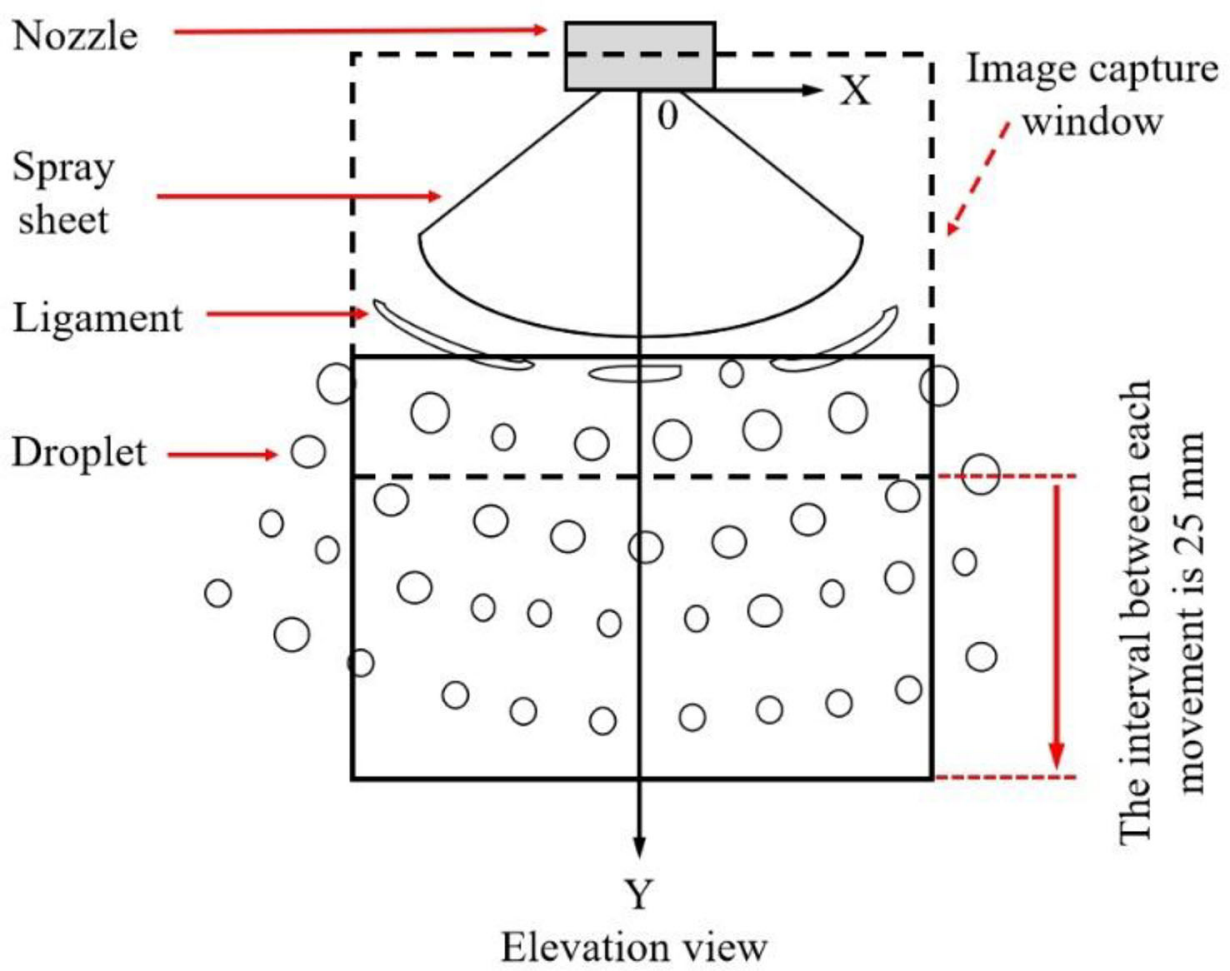
Diagram of spray coordinate and image capture window.

As indicated in **Figure 2**, the center point of the nozzle exit was defined as the origin of all coordinates; the right horizontal direction (spanwise direction) was set as the X direction; the vertically downward direction (streamwise direction) was set as the Y direction, and Z direction was perpendicular to the XOY plane. The images of spray along different Y distances were captured to analyze the spatial evolution of spray structures. Take **Figure 2** for example, the first image ① captured near the nozzle exit (as indicated with dash rectangle). Then, the second image ② was captured downstream with an interval of 25 mm (as indicated with solid rectangle). This process was repeated 10 times, which means that the spray structure in the range of 0~250 mm was captured. In the X direction, similar processes were used, and the distance interval is set as 10 mm.

To capture the transient spray structure, the exposure time of the high-speed camera was set as 2.16 μs. The frame rate was set to 2,000 fps (frames per second); therefore, the time interval between adjacent images is 0.5 ms. Under this condition, the resolution of image is 1,280 × 1,024 pixels. A ruler was used to measure the length of each nozzle before the experiment. A Commercial Image analysis software IPP (Image Pro Plus, Meyer Instruments, Inc., Houston, TX, USA) was used to measure the pixel length of the nozzle in the spray image. The ratio of the two measured length was defined as the scale factor SF (SF = length/pixel length).

### 2.3 Measurement of droplet size distribution

For water spray, the LD instrument and PDPA are commonly used methods to measure the droplet size distributions (Vieira et al., 2018; Sijs and Bonn, 2020). However, the oil-based emulsion solution is opaque, and the transmittance of its spray droplets is limited; therefore, the above methods are supposed to bring errors. The image processing method is more suitable for measuring those droplets that have relatively low transmittance (Zeng et al., 2015; Farzad et al., 2017; Patil et al., 2017).

**Figure 3:**
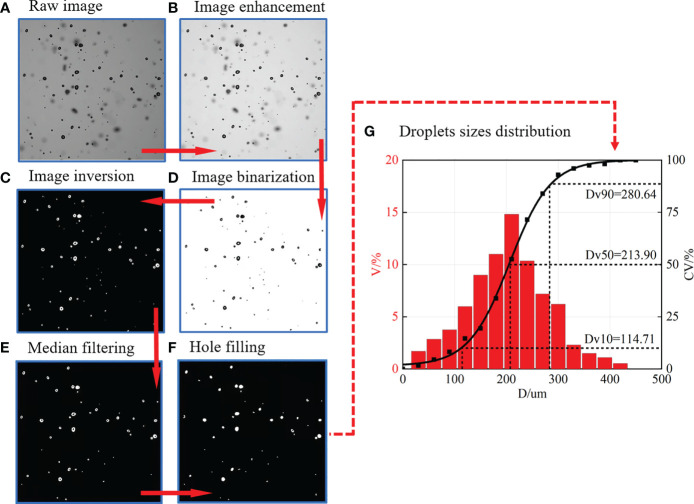
The methods of image processing and droplet size distribution measurement. **(A)** Raw image. **(B)** Image enhancement. **(C)** Image inversion. **(D)** Image binarization. **(E)** Median filtering. **(F)** Hole filling. **(G)** Droplets sizes distribution. V% denotes the volume percentage, CV% denotes the cumulative volume percentage.

In this paper, droplet sizes were measured on the basis of image processing. A typical image processing process was shown in **Figure 3**. The size of the captured sampling window was 10 × 10 mm. The raw image in **Figure 3A** was first enhanced on the basis of “Retinex” theory (Cai, 2020) to highlight the edge intensity gradient between the background and the target spray droplet. Then, the Otsu algorithm (Ostu, 1979) was used to determine the segmentation threshold, and the binary image was obtained (**Figure 3C**). After that, the image was inverted (**Figure 3D**), and the median filtering (Zhou et al., 2015) was used to remove noise. Finally, the imfill function (Zhou et al., 2015) was used to fill the “holes” within the droplets in **Figure 3E**. After image pre-processing, the “Bwlabel” function and “Regionprops” function (MATLAB, MathWorks Corporation, Natick, MA, USA) were used to detect and measure the spray droplets in **Figure 3F**. Next, an image batch processing code was independently developed on the basis of the commercial code MATLAB to calculate the volume size distribution of droplets. To reduce random error and ensure the enough samples, the numbers of images that are under the same experimental condition are processed and measured. In this paper, at least 100 images and 5,000 droplets were processed and measured for each experimental condition. One of the final results is presented in **Figure 3G**; the $D_{V}10$ , $D_{V}50$ , and $D_{V}90$ , which represent the diameter with cumulative volume less than 10%, 50%, and 90%, are commonly used to characterize droplet size in agricultural (ANSI/ASABE, 2020).

## 3 Results and discussion

### 3.1 Comparison between water and oil-based emulsion spray

**Figure 4:**
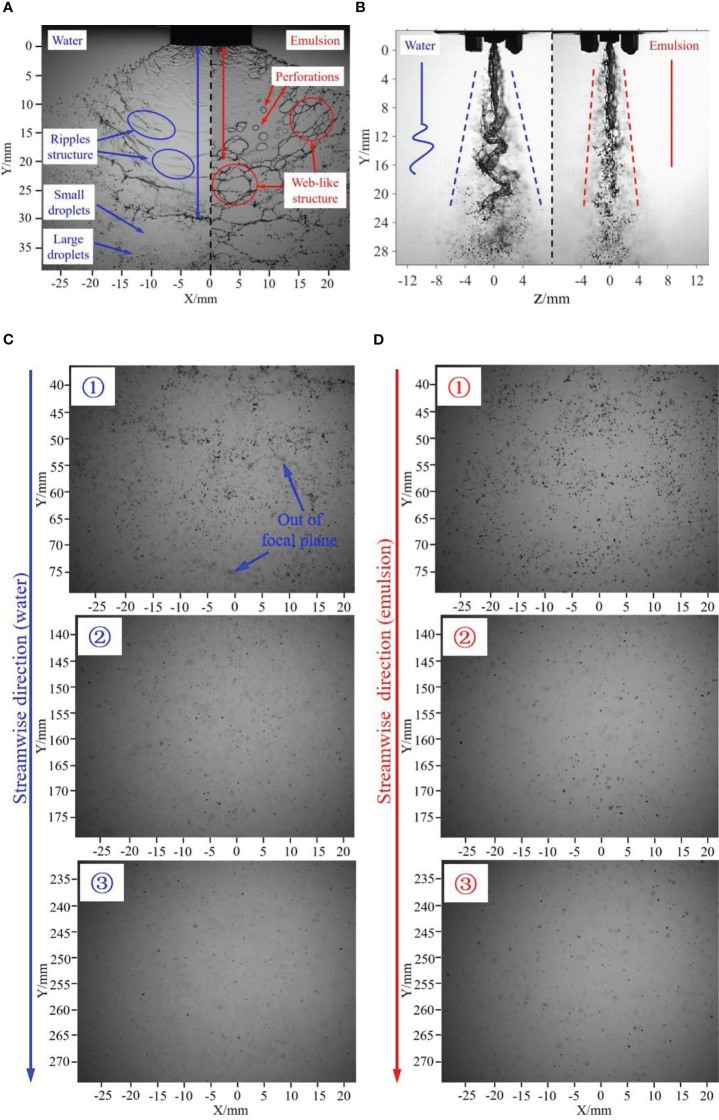
Comparison of water and emulsion spray. **(A)** Elevation view of water and emulsion spray sheet. **(B)** Side view of water and emulsion spray sheet. **(C)** Droplets images of water spray at different flow direction distances. **(D)** Droplets images of oil-based emulsion spray at different flow direction distances. The nozzle is ST110-03, spray pressure is 0.3 MPa, and the concentration of emulsion is 0.1%.

The spray sheets of water and oil-based emulsion are compared in **Figures 4A, B**. In the elevation view, for the water spray (the left half in **Figure 4A**), the liquid sheet has a relatively intact surface, with some “ripples” structures (marked by blue circle) on it. These ripples causes the breakup of the liquid sheet and the formation of the ligaments and droplets. As indicated in **Figure 4A**, this breakup regime causes both large and small droplets. For the oil-based emulsion (the right half in **Figure 4A**), it has a relatively shorter sheet length compared with water spray. Most notably, there are some perforations on the liquid sheet of oil-based emulsion spray. These perforations finally contact with each other to form web-like structure (marked by red circle) and breakup into droplets. The droplets generated by this breakup regime have a relatively uniform size distribution. In the side view, the water spray (the left half in **Figure 4B**) has a wave-like structure; on the contrary, the oil-based emulsion spray (the right half in **Figure 4B**) has no obviously fluctuation structures. As a result, the droplets of water spray distribute in a broad range in the Z direction.

The spray droplets of different Y positions are presented in **Figures 4C, D**. First, as indicated in phase ① in **Figure 4C**, the spray droplets have relatively bigger average size, and there are some droplets out of the focal plane for water spray. With the increase of flow direction distance, as shown in phases ② and ③ in **Figure 4C**, the average size of spray droplets obviously decreases; meanwhile, the number of droplets in the focal plane decreases. For emulsion spray (**Figure 4D**), it follows similar trend as water spray; however, the number of droplets that out of the focal plane is relatively smaller.

**Figure 5:**
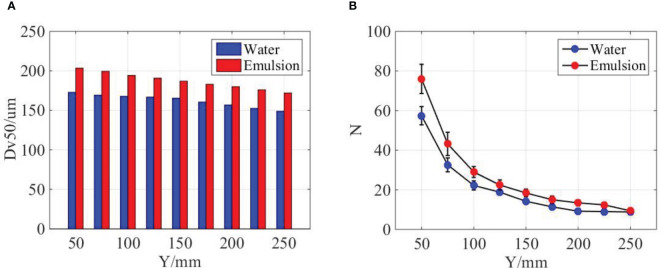
Comparison of water and emulsion spray. **(A)** The volumetric median diameter ( $D_{V}50$ ) of spray droplets at different streamwise distances. **(B)** The distribution density of spray droplets at different streamwise distances. The ST110-03 nozzle was used, the spray pressure is 0.3MPa, and the concentration of emulsion is 0.1%.

On the basis of the comparison above, it can be found that water spray is featured by wave-like structure, whereas the oil-based emulsion is featured by perforations. Different sheet structures cause different breakup regimes, and it supposes to produce different droplets distribution. Here, the quantitative information of droplet distribution of water and oil-based emulsion spray is discussed. Along the axis of symmetry, the volumetric median diameter ( $D_{V}50$ ) and distribution density (droplet number in the focal plane) of spray droplets at different streamwise distances are measured and compared in **Figures 5A, B**. As indicated in **Figure 5A**, for both water and oil-based emulsion spray, the droplet size decreases with the streamwise distance. The droplet sizes of oil-based emulsion spray are bigger than that of water spray at each positions. Meanwhile, measured results show that the size difference between water and oil-based emulsion spray decreases with the streamwise distance. For the distribution density of spray droplets, as shown in **Figure 5B**, in the range of 50~100 mm, both water and oil-based emulsion spray are dramatically decrease with streamwise distance. After Y = 200 mm, the distribution density changes little with streamwise distance, and the water and oil-based emulsion has no significant difference.

**Figure 6:**
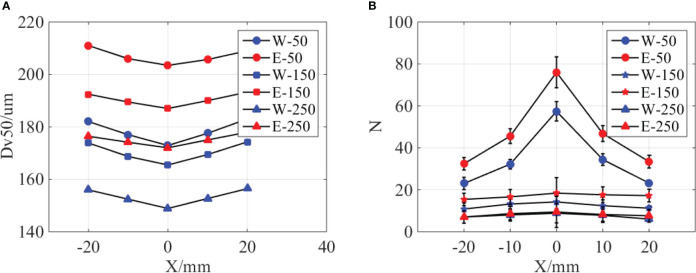
Comparison of water and emulsion spray. **(A)** The volumetric median diameter of spray droplets at different spanwise distances. “W-50” denotes water spray, and the position is 50 mm down below the nozzle exit. “E” denotes oil-based emulsion spray. **(B)** The distribution density of spray droplets at different spanwise distances. The ST110-03 nozzle was used, the spray pressure is 0.3MPa, and the concentration of emulsion is 0.1%.

The droplet size and distribution at different spanwise distances are measured and compared in **Figures 6A, B**. As indicated in **Figure 6A**, the size of water and oil-based emulsion spray follows a similar distribution, and the droplet size in middle position is smaller than lateral positions. At the same streamwise position, the droplet size of oil-based emulsion spray is larger than water spray. Meanwhile, the size difference between middle and lateral positions of oil-based emulsion spray is smaller than that of water spray. As for distribution density, as shown in **Figure 6B**, the droplet number in middle position is obviously larger than lateral positions. However, this difference will reduce with the increase of streamwise distance. When the streamwise distance increases to 250 mm, the distribution of water and oil-based emulsion spray has no significant different.

On the basis of the comparison above, it can be concluded that the volumetric median diameter of oil-based emulsion spray is bigger than water spray in both streamwise and spanwise direction positions. Oil-based emulsion spray also has a larger distribution density; however, the difference decreases with the increase of streamwise distance. From **Figure 4A**, it is know that the sheet length of oil-based emulsion spray is smaller than of water spray. The longer sheet length corresponds to the thinner sheet thickness under the same volume of flow. The thinner sheet is supposed to produce smaller droplets. This explains why water spray has a relatively smaller droplets sizes compared with oil-based emulsion spray. As indicated in **Figure 4B**, water spray has a wave-like motion in the Z direction. This fluctuation gives a kinetic energy in the Z direction for the droplet of water spray. As a result, water droplets distribute in a broad range in the Z direction and less droplets on the focal plane compared with oil-based emulsion spray. During the falling of the spray droplets, its kinetic energy decreases due to the resist of ambient air. Therefore, the difference between water and oil-based emulsion spray is reduced.

### 3.2 Effect of nozzle configuration on the droplet distribution of emulsion spray

**Figure 7:**
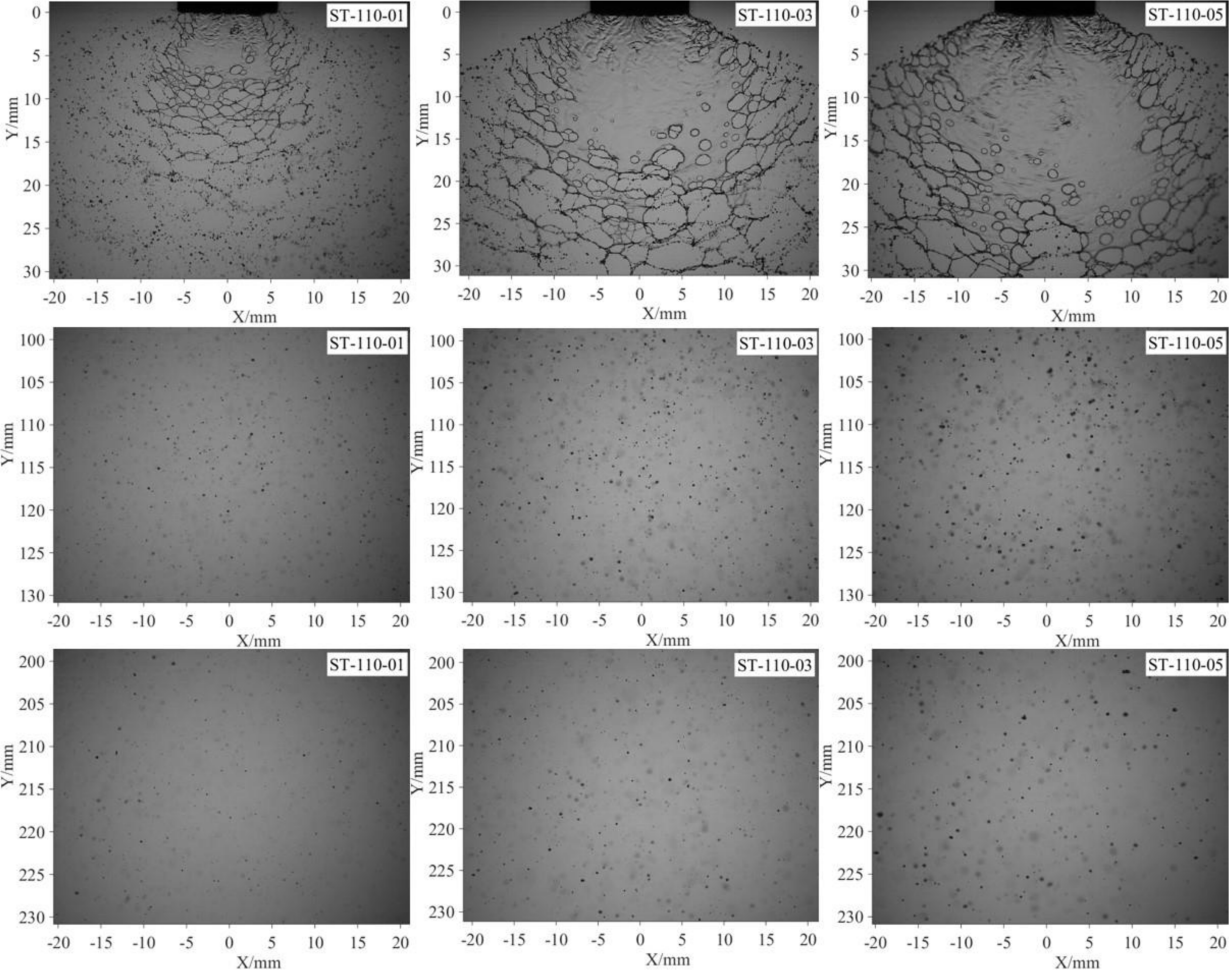
Spray images of different nozzle configurations. All the images are captured under the same pressure (0.3 MPa). The concentration of the oil-based emulsion is 0.1%.

Different nozzles are commonly used to modify the droplet size distribution. In this part, the effect of nozzle configuration on the droplet distribution of emulsion spray was discussed. First, the images of sheet structures and spray droplets of different nozzles are compared in **Figure 7**. As shown in the first line, nozzle configuration has a significant effect on the sheet structures. The sheet length is approximately 7 mm as the nozzle ST110-01 was used. With the nozzle changes from ST110-01 to ST110-03 and ST110-05, the sheet lengths increase to 18 and 28, mm respectively. Meanwhile, the sheet areas are obviously increased. For all the three spray sheets, there are some perforations on them, which indicate that nozzle configurations do not change the breakup regime. As shown in the second and third lines, the droplets generated by the three nozzles have different size, and the droplet of nozzle ST110-05 has bigger size at all the streamwise positions. For the oil-based emulsion spray, the perforations cause the breakup of the liquid sheet. For nozzle ST110-01, the position that perforation generation is closer to the nozzle exit. Therefore, it has a shorter sheet length and smaller sheet area. As shown in **Figure 1B**, the discharge orifice of nozzle ST110-05 has a bigger size, and it supposed to produce thicker spray sheet. The generation position of perforations is far from the nozzle exit; as a result, it has a longer sheet length and larger sheet area.

**Figure 8:**
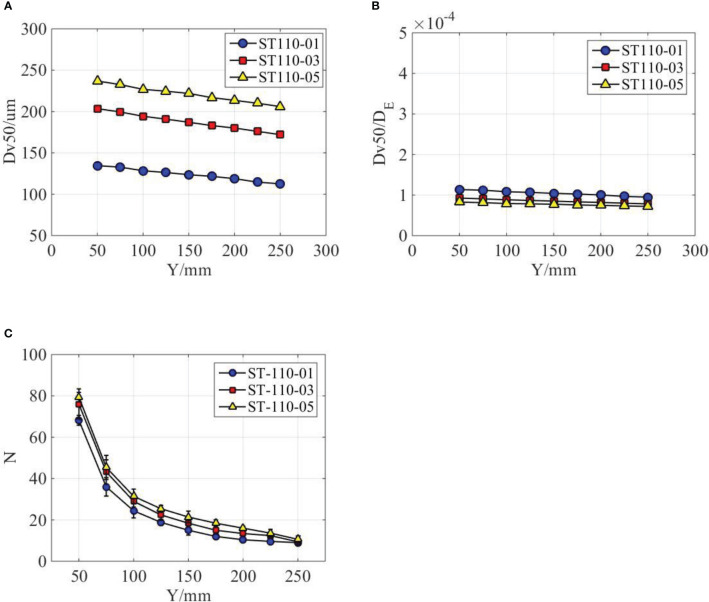
Droplet distribution characteristics at different nozzle configurations. **(A)** The effect of nozzle configurations on the volumetric median diameter. **(B)** Fitting curves of different nozzle configurations. **(C)** The effect of nozzle configurations on the droplet distribution density.

More quantitative information of spray droplets is presented in **Figure 8**. First, as indicated in **Figure 8A**, with the increase of streamwise distance, the droplets sizes are gradually decrease for all the three nozzles. The nozzle configuration does not change the evolution patterns but has a significant effect on the droplet size. As the nozzle type is changed from ST110-01 to ST110-03 and ST110-05, the $D_{V}50$ increases to 51.19% and 76.00%, respectively. Different nozzles have different exit sizes (as shown in **Figure 1B**); therefore, we try to nondimensionalize the droplet sizes by using equivalent diameter of nozzle exits, as indicated in **Figure 8B**. Interestingly, we found that the data of different nozzles tend to coincide. It is indicated that the droplet size of oil-based emulsion spray can be scaled by the size of nozzle exit. A possible reason responsible for this interesting finding is that the size of spray droplets was determined by the thickness of spray sheet, which was controlled by the size of nozzle exit. For the nozzles used in this paper, the volumetric median diameter is approximately 1/10,000th of the equivalent diameter of nozzle exit. For the distribution density of spray droplets, as shown in **Figure 8C**, it is slightly increased with the nozzle type changes from ST110-01 to ST110-03 and ST110-05. Clearly, nozzle configuration has little effect on the distribution density of spray droplets.

### 3.3 Effect of emulsion concentration on the droplet distribution of emulsion spray

**Figure 9:**
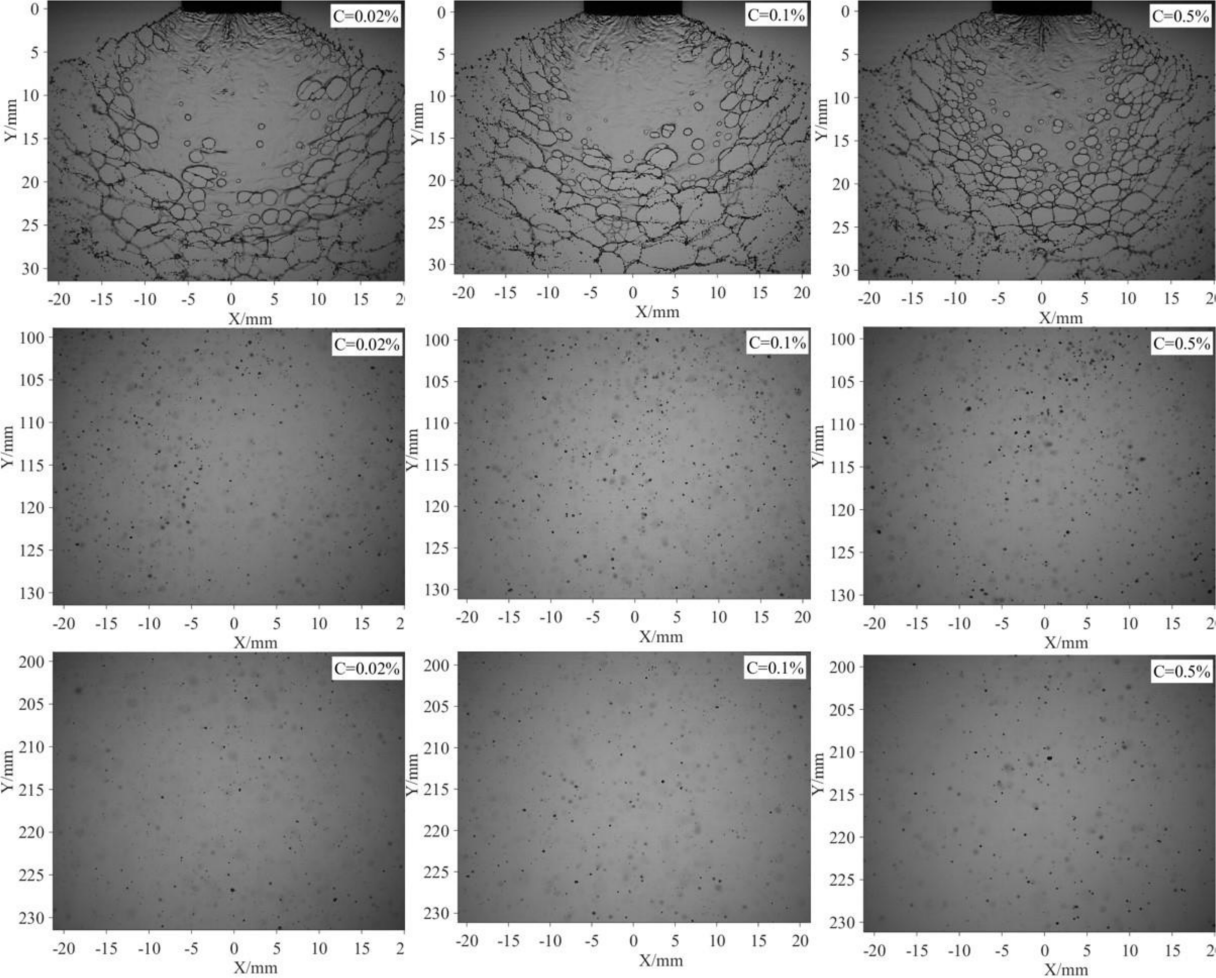
Spray images of different emulsion concentrations. All the images are captured under the same pressure (0.3 MPa) and same nozzle (ST-110-03).

The spray images of different emulsion concentrations are compared in **Figure 9**. As indicated in the first line, with the increase of emulsion concentration, the number of perforations on the spray sheet is obviously increased. The development of the perforations forms web-structure and causes the breakup of liquid sheet. As a result, the sheet of higher emulsion concentration has a shorter length and smaller area. As indicated in the second and third lines, the droplet distributions have no significant different. Clearly, the effect emulsion concentration on the spray droplets is limited compared with nozzle configurations.

**Figure 10:**
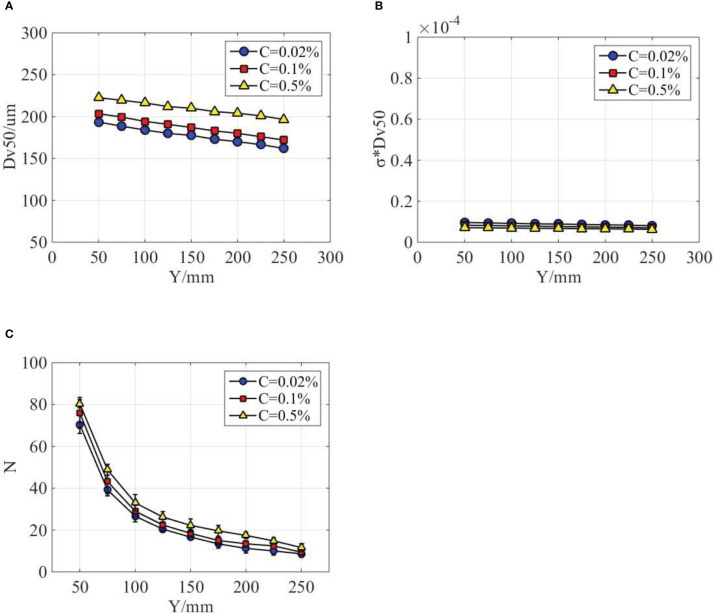
Droplet distribution characteristics at different emulsion concentrations. **(A)** The effect of emulsion concentrations on the volumetric median diameter. **(B)** Fitting curves of different emulsion concentrations. **(C)** The effect of emulsion concentrations on the droplet distribution density.

The droplet size and distribution of different emulsion concentrations were measured and compared in **Figure 10**. As indicated in **Figure 10A**, the volumetric median diameters generally increased with the increase of emulsion concentration. It increases by 5.17% as the emulsion concentration increases from 0.02% to 0.1%. However, when the emulsion concentration increases from 0.1% to 0.5%, the volumetric median diameters increased by 9.39%. The volumetric median diameters gradually decreased with the increase of axial distance for all the three emulsion concentrations. However, the decrease rate for emulsion concentration of 0.5% is relatively smaller. With the increase of emulsion concentration, the surface tension of the spray liquid decrease. If the product of volumetric median diameter and corresponding surface tension was used, we found that the data of different emulsion concentrations tend to coincide, as shown in **Figure 10B**. A possible reason responsible for this phenomenon is that the droplets sizes can be modified by the surface tension of spray liquid. Some existing references (Hilz and Vermeer, 2013) indeed verified that the size of spray droplets was inversely proportional to surface tension of spray liquid. From **Table 2**, it can be found that surface tension decreased with the increase of emulsion concentration. The droplet distribution density of different emulsion concentrations is presented in **Figure 10C**. The droplet number generally increased with the emulsion concentrations; however, the difference of three emulsion concentrations is limited.

## 4 Conclusions

In this paper, the spatial sheet structure and droplet distribution of oil-based emulsion spray are captured and measured. The effect of nozzle configuration and emulsion concentration of the sheet structure and droplet distribution were discussed. Major conclusions drawn from the study are as follows.

Oil-based emulsion spray has a significantly different atomization mechanism compare water spray. Water spray is featured by wave atomization mechanism, whereas the oil-based emulsion spray is featured by perforation atomization mechanism. With the presence of holes, the spray sheet breaks earlier; therefore, the droplet size is larger than that of the water spray in both streamwise and spanwise direction. Because of the wave-like fluctuation of water spray, water droplets disperse in a broad range compared with oil-based emulsion spray. This spatial distribution difference between water and oil-based emulsion spray will reduce due to the resist of ambient air.

Nozzle configuration does not change the atomization mechanism of oil-based emulsion spray; however, it has a significant effect on the spray droplet size. The nozzle with a larger discharge orifice produces longer sheet length, larger sheet area, and bigger droplets. Interestingly, we found that the spray droplet size can be scaled by the equivalent diameter of discharge orifice of nozzles. For the nozzles used in this paper, the volumetric median diameter is approximately 1/10,000th of the equivalent diameter. The effect of nozzle configuration on the spatial distribution of the spray droplets is limited.

The typical structure, perforations, is sensitive to the variation of emulsion concentration. With the increase of emulsion concentration, the number of perforation on the spray sheet increased; as a result, sheet length and area decreased. The effect of emulsion concentration on the droplet size is limited compared with nozzle configuration. If the product of volumetric median diameter and corresponding surface tension was used, then it is found that data of different emulsion concentrations tend to coincide.

## Data availability statement

The original contributions presented in the study are included in the article/supplementary material. Further inquiries can be directed to the corresponding author.

## Author contributions

CG: Methodology, Conceptualization; FC: Writing-Original draft preparation; BC: Capture of spray images; AW: Image processing; ZZ: Statistical analysis of the results; ZJZ: Writing Review and Editing; LY: Revised and Editing. All authors contributed to the article and approved the submitted version.
